# The Influence of Gas–Wall and Gas–Gas Interactions on the Accommodation Coefficients for Rarefied Gases: A Molecular Dynamics Study

**DOI:** 10.3390/mi11030319

**Published:** 2020-03-19

**Authors:** Shahin Mohammad Nejad, Silvia Nedea, Arjan Frijns, David Smeulders

**Affiliations:** Energy Technology, Department of Mechanical Engineering, Eindhoven University of Technology, 5600 MB Eindhoven, The Netherlands; s.mohammad.nejad@tue.nl (S.M.N.); s.v.nedea@tue.nl (S.N.); d.m.j.smeulders@tue.nl (D.S.)

**Keywords:** rarefied gas, accommodation coefficient, molecular dynamics (MD) simulation, Ar–Au interaction, He–Au interaction, mixing rules, ab-initio potentials

## Abstract

Molecular dynamics (MD) simulations are conducted to determine energy and momentum accommodation coefficients at the interface between rarefied gas and solid walls. The MD simulation setup consists of two parallel walls, and of inert gas confined between them. Different mixing rules, as well as existing ab-initio computations combined with interatomic Lennard-Jones potentials were employed in MD simulations to investigate the corresponding effects of gas-surface interaction strength on accommodation coefficients for Argon and Helium gases on a gold surface. Comparing the obtained MD results for accommodation coefficients with empirical and numerical values in the literature revealed that the interaction potential based on ab-initio calculations is the most reliable one for computing accommodation coefficients. Finally, it is shown that gas–gas interactions in the two parallel walls approach led to an enhancement in computed accommodation coefficients compared to the molecular beam approach. The values for the two parallel walls approach are also closer to the experimental values.

## 1. Introduction

Rarefied gas condition is encountered in a broad range of modern engineering applications; for example, in low pressure devices such as semiconductor manufacturing and spacecraft flying at high altitudes, as well as small-scale structures such as microelectronic devices and micro/nanoelectromechanical systems (M/NEMS) [1,2]. In all these applications to achieve the effective thermal management, a fundamental understanding of gas–surface interactions (GSI) is of paramount importance. The degree of rarefaction of a gas is quantified by Knudsen number ($\mathrm{Kn}=\frac{\lambda }{L}$), where λ is the mean free path of the gas molecule and L is the characteristic length scale. It is known that a gas is regarded as rarefied if Kn > 0.01 [1]. Over the years, a wide range of experimental [3,4,5] and numerical studies [6,7,8] have been carried out, investigating rarefied gas-solid surface interactions. Due to the complexities involved in the instrumentation, an empirical study of GSI is a very challenging and time-consuming task. Regarding computational techniques, due to the noncontinuum gas behavior adjacent to the solid surface, common continuum approaches (Navier–Stokes equations) are not applicable to describe energy and momentum exchange at GSI. Herein, particle-based simulations methods such as MD simulations [9] are considered a promising candidate to study GSI. MD simulations can provide an atomistic-level understanding of the scattering dynamics of the gas molecules interacting with solid surfaces. Energy and momentum accommodation coefficients (E/MACs), which are the most relevant parameters involved in GSI models, describe the degree at which a gas attains its thermal or kinematic equilibrium with a surface while interacting with it. MD simulation is a very promising tool to determine different accommodation coefficients. These coefficients can be fed into semi-empirical GSI models such as Maxwell’s model [10] or Cercignani–Lampis–Lord (CLL) model [11] that can be employed as boundary conditions for higher-scale simulation techniques such as Direct Simulation Monte Carlo (DSMC) [12], Lattice Boltzmann method (LBM) [13], and method of moments (MoM) [14] to describe heat and mass flow at macroscopic level under rarefied condition. In literature there are various numerical studies in which MD simulations are employed to determine accommodation coefficients for different gas-solid surface combinations [15,16,17,18,19,20,21,22]. The general objective of all such investigations is to find the correlation between the energy and momentum accommodation coefficients and input parameters such as the gas temperature or purity, gas molecular weight (MW), surface condition (i.e., surface roughness, cleanness, temperature and chemistry), as well as the gas–surface interaction strength. Briefly summarized, results in the literature reveal that E/MACs decrease by increasing the temperature and implicitly the kinetic energy of the molecules. Moreover, increasing the surface roughness, gas molecular mass, and gas–solid interaction strength lead to an increase in E/MACs. Gas molecules approaching a surface are sometimes trapped by the potential well and stager on the surface for a while as they are physically adsorbed. The gas molecules may escape the potential well after some residence time through which they lose sufficient amount of their thermal or kinetic energy such that they accommodate to the surface temperature at a higher degree (i.e., resulting in a higher accommodation coefficient). This phenomenon called trapping-desorption is more likely to happen at lower temperature, higher surface roughness and, higher gas MW, as well as stronger gas-solid interaction which at the end causes higher E/MACs at aforementioned conditions. 

Due to the superposition of many factors affecting the phenomenon of gas-surface interaction, sometimes in literature for the same pair of gas–solid surface, a considerable discrepancy in the values of accommodation coefficients, obtained by different MD simulation approaches is found. As an example, for the Platinum–Argon combination the obtained values for the tangential momentum accommodation coefficient by Yamamoto et al. [23] and Hyakutake et al. [24] were 0.19 and 0.89, respectively. Accurate values of accommodation coefficients are essential for the better assessment of the overall transport properties of rarefied gases. Therefore, we will compare most common MD approaches and study the effect on the resulting thermal and momentum accommodation coefficients, and compare the results with experimental values. 

In most previous MD simulations [19,21,22] to compute E/MACs, the molecular beam approach was employed, in which the gas molecule adjacent to the surface is assumed to interact only with the wall atoms, and its initial velocity is sampled from an equilibrium distribution at certain temperature. Such assumptions are valid for a highly-rarefied gases (i.e., Kn > 10). However, the condition is very different in most M/NEMS applications, where less degree of rarefaction is encountered (Kn < 1) [1]. In such systems, in the case of a temperature difference between gas and surface, gas molecules will experience non-equilibrium processes. Furthermore, both of gas–wall and gas–gas interactions are equally important.

In the present work, two parallel plates MD approach is applied to calculate E/MACs of noble gases (Ar and He) interacting with Gold (Au) surface. Firstly, the dependence of E/MACs on the gas pressure in the system was investigated, which to our best knowledge has not been studied by MD simulations, previously. The impact of gas-solid interatomic potential on E/MACs was also characterized. To do so, a pairwise Lennard-Jones (LJ) potential was considered at the solid-gas interface. The LJ potential parameters were computed using different approximation methods such as Lorentz–Berthelot (LB) and Fender–Halsey (FH) mixing rules, as well as taking form existing ab-initio calculations. It has been observed that an interaction potential based on quantum calculations such as ab-initio computations is the most reliable one for computing different ACs. Such a behavior has been reported by Mane et al. [21] and Daun et al. [25], where using molecular beam approach, they studied the interaction between monoatomic gases with aluminum and iron surfaces, respectively. At the end, to unravel the importance of including gas–gas interactions on the MD obtained accommodations coefficients, we compare our two parallel plates results with those obtained by the molecular beam approach.

## 2. Materials and Methods 

### 2.1. Molecular Dynamics (MD) Simulations

**Figure 1:**
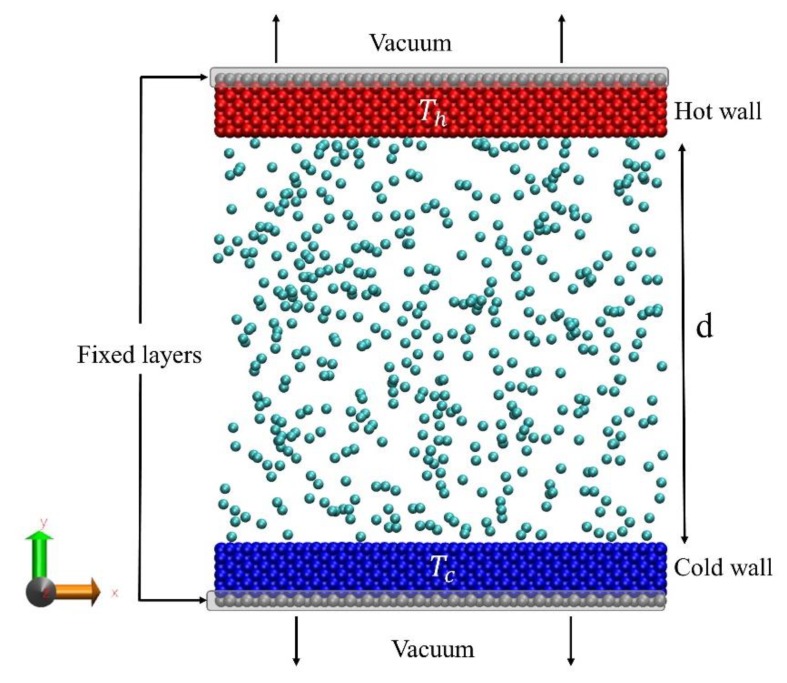
Schematic representation of the simulation setup; two walls kept at a distance d apart; they are thermostated at a low temperature T_c_ (bottom wall) and a high temperature T_h_ (top wall).

The MD simulation setup considered in this work is a three-dimensional system, in which a monatomic gas is confined between two parallel walls (see Figure 1).

The cross-section area of the walls is 10 by 10 nm, and each of them consists of 8750 gold atoms arranged in a FCC structure. The walls are separated from each other in the y direction. The temperatures of the bottom and top walls are maintained at T_c_ = 300 K and T_h_ = 350 K, respectively, using Berendsen thermostats. In each wall, the outermost layer is fixed to prevent the wall from any translational or rotational motion. Periodic boundary conditions are applied in all three directions. Since the simulation box is also periodic in y direction, the walls are not placed directly at the periodic boundary. Vacuum has been considered between the walls at the periodic boundary in order to prevent direct contact and heat conduction between the hot and cold walls. At the beginning of the simulation, the temperature of the gas molecules is set at the 300 K, which corresponds to the root mean square velocity (V_rms_) of 423 and 1367 m/s, for Ar and He gases, respectively. Herein, the gas molecules are not coupled to an external heat bath, and their temperature change is caused only via collisions with other particles (gas and solid particles). As it will be discussed in detail in the proceeding section, in order to obtain reliable results for E/MACs, a large number of collisions between gas molecules and the wall surface are required: 100,000 collisions have been considered here, as it has been recommended in previous studies [15,18] for a similar simulation setup. Increasing the number of collisions can be achieved by either extending the walls surface area or increasing the gas number density. Considering the computational time, the latter approach is more favorable. On the other hand, higher gas number density means higher pressure and the possibility to surpass the gas critical pressure, which is not desirable here, since the properties of a supercritical fluid differs in many aspects from a gas. In this work, in order to increase the gas number density, walls are positioned in the closest distance from each other in such a way that the pressure in the system does not exceed the critical pressure (P_cr_) value in the system (For Ar: P_cr_ = 4.86 MPa, and for He: P_cr_ = 0.23 MPa [26]). This results in a separation distance of d = 11 nm and d = 102 nm for Ar and He gases, respectively. The Knudsen number in the case of the system with Ar (P_Ar_ = 2.75 MPa) and He (P_He_ = 0.21 MPa) gases are 0.23 and 0.56, respectively.

The interaction between Au atoms are described by the embedded atom model (EAM) potential [27], whereas the gas–gas and gas–solid interactions are modeled by the Lennard-Jones (LJ) potential. Herein, noble gas-Au pair potential coefficients have been calculated by commonly used (LB) mixing rule:

(1) $$\sigma_{\mathrm{ij}}=\frac{\sigma_{\mathrm{ii}}+\sigma_{\mathrm{jj}}}{2}\;,\;\varepsilon_{ij}=\sqrt{\varepsilon_{ii}\varepsilon_{jj}}$$

and (FH) mixing rule:

(2) $$\sigma_{\mathrm{ij}}=\frac{\sigma_{\mathrm{ii}}+\sigma_{\mathrm{jj}}}{2}\;,\varepsilon_{ij}=\frac{2\varepsilon_{ii}\varepsilon_{jj}}{\varepsilon_{ii}+\varepsilon_{jj}}$$

as well as existing ab-initio pair potentials [22,28], derived from computational quantum mechanics. 

The detailed gas-gas and gases-Au interatomic potential parameters used for mixing rules are presented in Table 1. In addition, the pair potential coefficients based on existing ab-initio calculations are listed in Table 2. The LJ cut-off distance (r_c_) for gas-gas interactions is set at 2.5 times the LJ length parameter (*σ_ii_*). In addition, for both of Au-Ar and Au-He pairs we used r_c_ = 12 Å which is similar to the cut-off radii that were used in the ab-initio simulations [22]. 

For implementing MD simulations, LAMMPS molecular dynamics package was used [31]. All simulation setups were initially equilibrated for 1 ns (time step = 1 fs) for Ar and 3 ns for He. Afterwards, the production run was started and proceeded for the next 20 and 60 ns for Ar and He cases, respectively.

### 2.2. Computing Accommodation Coefficients 

**Figure 2:**
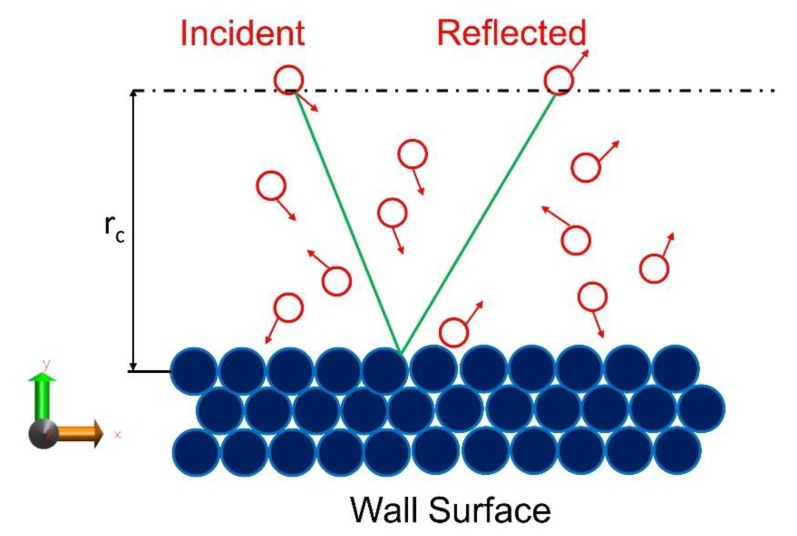
Schematic of gas-surface interaction used to compute accommodation coefficients in molecular dynamics (MD) simulations. Here the green line is a simplified example of gas molecule trajectory. The virtual border for the computation of the accommodation coefficients is placed at distance r_c_ = 12 Å from the solid surface.

To compute E/MACs using MD simulations, the trajectory of each gas molecule in the simulation box is monitored during the specified simulation time. Herein, the collision is defined in such a way that if a gas molecule approaching the surface crosses a virtual border placed in certain distance from the surface, its velocity components are recorded. For the same particle after re-emitting from the surface, when it reaches back to the virtual border, its velocity components are recorded again. Both scattered and trapped particles that are desorbed from the wall are considered here. As it is depicted in Figure 2, in this study the virtual border is located at one gas-wall interaction cut off distance (r_c_ = 12 Å) away from the wall surface in order to guarantee that the gas molecule is not affected by the wall potential. 

The accommodation coefficients were calculated by the least-square method proposed by Spijker et al. [18]. In this approach, which is based on the correlation between input (Impinging) and output (Reflected) data obtained from MD simulations, the AC (α) can be computed as follows:

(3) $$\alpha_{q}=1-\frac{\sum_{i}(Q_{I}^{i}-\langle Q_{I}\rangle )(Q_{R}^{i}-\langle Q_{R}\rangle )}{\sum_{i}{\left(Q_{I}^{i}-\langle Q_{I}\rangle \right)}^{2}},$$

where subscript q can be any kinematic quantity, such as the gas molecule velocity in a certain direction (for momentum accommodation) or its total kinetic energy (for thermal energy). Q_I_ and Q_R_ referred to the considered quantity for the impinging and reflected particles, respectively. To be more specific, when a gas atom approaching to the surface (v_y_ < 0) passes through the virtual border (dotted line in Figure 2) it will classified as an impinging particle. From the other side, when a gas atom going outwards with respect to the surface (v_y_ > 0) passes through the virtual border it will be considered as reflected particle. The bracket notations denote that the average value for these quantities is calculated.

## 3. Results and Discussion

**Figure 3:**
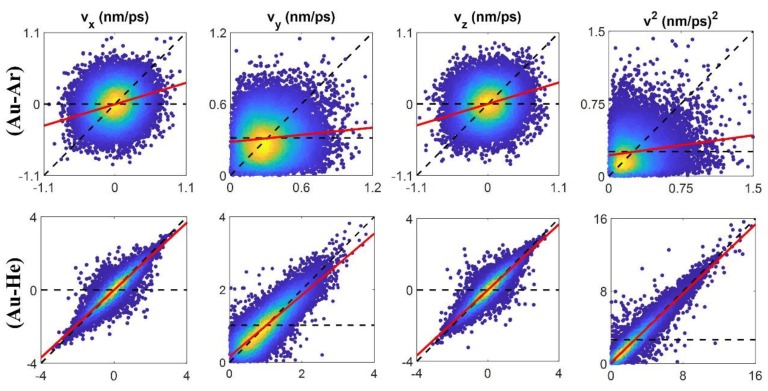
Velocity correlations of impinging (x-axis) and reflected (y-axis) velocity components of Ar and He on Au surface at 300 K using ab-initio potential. The dashes horizontal and diagonal lines indicate fully diffusive and specular conditions, respectively. The red line refers to the linear fit of the collision data obtained by MD simulations.

In order to compute E/MACs, after gathering collision data from MD simulation, the correlation between relevant impinging and outgoing velocities was studies. These correlations can be illustrated as a two-dimensional probability distribution profile, which for a particular impinging velocity gives the distribution of reflected velocities (see Figure 3). Herein, a line is fitted to the collision data based on least-square approximation (red line in Figure 3). Comparing the slope of this line, which is actually the fractional part of Equation (3), with dashed horizontal (fully diffusive) and diagonal (fully specular) lines gives us the accommodation coefficient.

First of all, the dependency of computed E/MACs on gas pressure between two walls was investigated. Herein, ab-initio pair potential was employed to describe gas-wall interactions. By decreasing the number of gas atoms in the simulation box, the pressure was reduced from 2.75 MPa to 0.42 MPa, and from 0.21 to 0.04 MPa in the case of Ar and He, respectively. As it is depicted in Table 3, for both gases, decreasing pressure does not have a significant impact on obtained accommodation coefficients, but considerably increases the MD simulation time required to record the same number of collisions. Similar pressure dependency has been also reported in an experimental study by Thomas and Brown [32]. Therefore, in the remaining part of this paper we perform our simulations at the pressures of 2.75 and 0.21 MPa for Ar and He gases, respectively.

**Figure 4:**
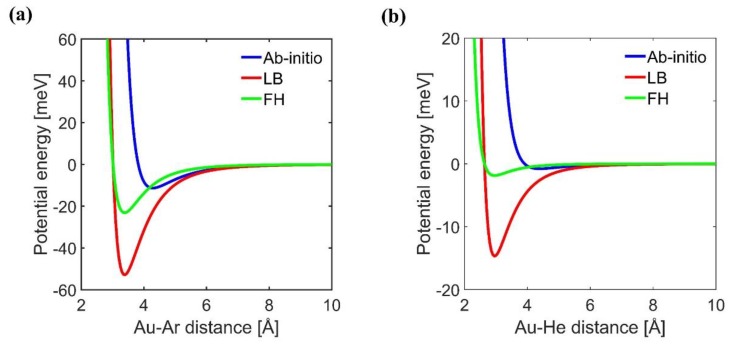
Pair potential energy plots of noble gases interaction with Au surface: (**a**) Au–Ar; (**b**) Au–He.

**Figure 5:**
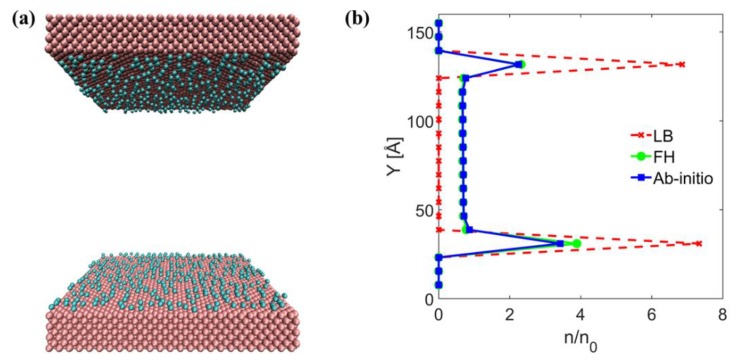
(**a**) Adsorption of Ar molecules on Au surface based on pair potential obtained from LB mixing rule; (**b**) normalized number density for different Au–Ar interaction potentials.

In the next step, the comparison between gas-wall interaction potentials obtained using different methods is shown in Figure 4. This figure shows that the FH mixing rule is relatively softer than the LB mixing, and that the ab-initio potential is softer than both mixing rules. Furthermore, it is depicted that the LB mixing rule highly overestimates the potential well depth. Such an overprediction in the case of a heavy gas like Ar causes that during MD run all Argon atoms are adsorbed on the solid surfaces (see Figure 5a), and that they do not leave the surface anymore. Therefore, for none of the impinging gas particles an outgoing velocity can be recorded, and E/MAC values are numerically unobtainable. In addition, the normalized number density distributions in the case of aforementioned system using varied interaction potentials are depicted in Figure 5b. Herein, initially it can be understood that the gas density adjacent to the wall surfaces is higher than the bulk density (n_0_) in the central part of the system: stronger is the gas-wall interaction the higher is the density profile peak near to the wall. This is in agreement with the behavior reported in [33]. Furthermore, in the case of the LB potential, except in the vicinity of the walls, the gas density goes to zero.

The obtained E/MACs for the aforementioned systems at the bottom wall (T_w_ = 300 K) are reported in Table 4. The reason why only E/MACs on bottom wall are reported here is for a further comparison with experimental data which are in the same temperature range. As it is shown in [15,18], the temperature gradient between two walls causes only a minor reduction in obtained values for E/MACs on the bottom wall. Therefore, this can be neglected. However, in order to resemble the experimental two parallel plates, in which the presence of a small temperature gradient (T_h_ − T_c_ << T_c_) is a must [34], a temperature difference was imposed between the two plates in our MD simulations. For both noble gas cases, E/MACs increase with increasing the potential well depth. This is in agreement with the behavior as reported in other numerical studies [16,21]. The reason lies in the fact that higher gas–wall interaction strength increases the likelihood of trapping-desorption phenomenon, which at the end causes higher energy and momentum exchange between the solid surface and the neighboring gas. In the case of Au–Ar, the reported empirical value for EAC (α_E_) using two parallel plates approach at 296 K is 0.85 [34], which is in excellent agreement with obtained value for EAC using the potential based on ab-initio computations for our parallel walls assembly. Furthermore, Agrawal and Prabha [35] have reported that the tangential-MAC (TMAC) for Ar on commonly employed surface materials is 0.893, which this value also is consistent with obtained results for TMAC in different directions (α_x_ and α_z_) in our case based on ab-initio pair potential. The EAC using FH mixing rule is 0.913, which is slightly higher than the value obtained by ab-initio potential.

For Au–He, Trott et al. [34] have measured EAC = 0.31 at 296 K, which is higher than all values reported in Table 4 for the same combination. To elucidate the possible reason behind the observed mismatch, it is noteworthy to mention that the surface roughness and the type of premeasurement treatment employed to clean the surface under investigation have significant impacts on obtained experimental results for E/MACs. For instance, for He–Pt pair at room temperature Mann [36] reported EAC = 0.03. For the same combination, in another experimental study [37] the measured EAC at 303 K was 0.238. The most important difference between the two aforementioned studies is that in the first study the metal surface is perfectly clean, but in the latter case the metal surface is somehow contaminated and it is also partially covered by testing gas. In addition, in another experiment [38] using Tungsten as the substrate, it was reported that EAC for He can vary from 0.017 (clean surface) to 0.23 (untreated surface). In Reference [34] it is also mentioned that recontamination of the surface is highly possible after the surface treatment technique that they used. Considering that in the performed MD simulation the surface is assumed to be atomistically smooth and clean, it can be inferred that MD results for E/MACs at the first place should be compared with experimental results on very clean and pure solid surface. Herein, assuming that clean Au surface has a similar behavior as clean Platinum and Tungsten surfaces, it is seen that the obtained results for EAC of Au–He using pair potential based on both FH mixing rule and ab-initio computations are similar to the values reported in [36] and [38] for He on clean Pt and Tungsten surface, respectively. 

**Figure 6:**
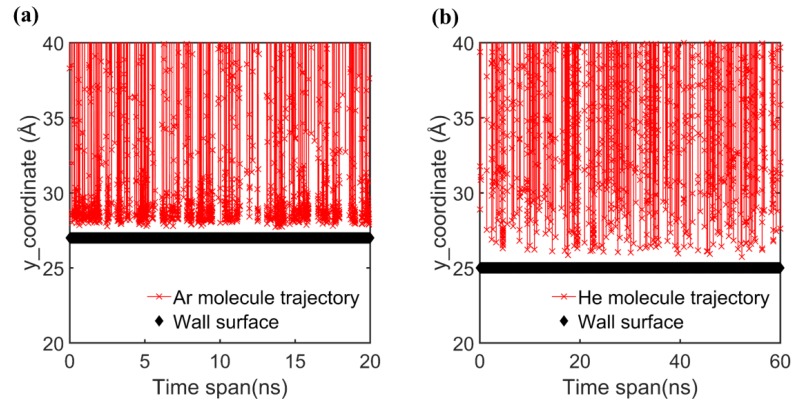
Trajectories of noble gases in vicinity of Au surface. (**a**) Ar–Au pair; (**b**) He–Au pair. Red crosses depict the y-coordinate of gas atoms as a function of simulation time.

The presence of contamination on a real surface causes the accumulating of gas molecules on the surface which at the end leads to measuring higher E/MACs. To support this, the trajectories of Ar and He molecules interacting with Au surface using ab-initio computations pair potential during our MD simulation are depicted in Figure 6. In this figure the red crosses indicate the y-position of each gas molecules as function of simulation time. When an accumulation of red crosses is shown in certain area, it means that gas atom remains for a longer time in that area. It is shown that in the case of Ar molecule, which has higher molecular weight ($\frac{{\mathrm{MW}}_{\mathrm{Ar}}}{{\mathrm{MW}}_{\mathrm{He}}}≃10$) and stronger interaction with Au surface, higher number of multiple collisions with Au surface has occurred (accumulation of red dots in vicinity of Au surface for Au–Ar pair). This means that in MD simulation of Au–Ar pair, there is higher chance that we see a layer of gas molecules adjacent to the solid surface. This layer of gas molecules can resemble the contamination in the case of experimental study, in which achieving a real clean surface is very challenging. Due to the presence of this layer on the surface in the case of Au–Ar combination, gas–gas interactions near the wall are more frequent. Since the gas–gas interaction strength is typically higher than gas-wall interaction strength (see Table 1 and Table 2), it can be deduced that forming a gas layer at vicinity of surface leads to deriving higher values for E/MACs by MD simulations. Therefore, it is more likely that E/MACs obtained by MD simulations for a heavy argon gas match the experimental results.

Liao et al. [22] computed also E/MACs for Ar–Au and He–Au pairs using a molecular beam approach based on the same ab-initio potential that was employed here. As it is shown in Table 4, their results for E/MACs are lower than those obtained in this study. In the case of molecular beam approach gas molecules adjacent to the surface are considered to interact only with wall atoms, and gas-gas interactions are ignored. While in the case of the parallel plates approach due to presence of other gas molecules in the system, gas particles will be partly reflected back by other particles to the surface resulting in relatively more gas–wall interactions per molecule and therefore resulting in an increase in thermal and momentum accommodation coefficients. 

## 4. Conclusions

The energy and momentum accommodation coefficients of monoatomic gases (Ar and He) on gold surface were determined through MD simulations. Initially, the impact of gas pressure on computed accommodation coefficients was investigated. Reducing gas pressure has a minor influence on accommodation coefficient values, whereas it can considerably increase the MD simulations time. 

The effect of gas-wall interaction strength on accommodation coefficients was studied for different mixing rules, as well as for ab-initio pair potential. It was concluded that larger energy well depths in potential energy function leads to higher accommodation coefficients. For Ar–Au and He–Au gas-wall interactions, the energy well depth is overestimated for both Lorentz–Berthelot and for Fender–Halsey mixing rules resulting in an overestimation of the E/MACs. In the case of Ar–Au, the Lorentz–Berthelot mixing rule highly overestimates the potential well depth. This issue causes a fully saturated solid surface in which computing the accommodation coefficients from numerical point of view is not possible. It has been found out that the accommodation coefficients obtained by pair potential based on ab-initio computations are always in a reasonable agreement with experimental and numerical results in the relevant literature.

Comparing the obtained results for accommodation coefficients in this work with another study in which a molecular beam MD approach was used to compute accommodation coefficients reveals that gas–gas interaction is an important aspect that needs to be taken into account in the transient Knudsen regime since it leads to an enhancement in obtained accommodation coefficients. 

## Figures and Tables

**Table 1:** Gases-gold interaction potential parameters used by mixing rules and molecular weights.

Atom Type	*ε_ii_* (meV)	*σ_ii_* (Å)	MW (a.m.u)
Au [29]	229.4	2.63	196.96
Ar [30]	12.2	3.35	39.94
He [30]	0.94	2.64	4.00

**Table 2:** Gases-gold interaction potential parameters based on ab-initio computations [22].

Parameter	Value
$\varepsilon_{\mathrm{Au}-\mathrm{Ar}}$	11.36 (meV)
$\sigma_{\mathrm{Au}-\mathrm{Ar}}$	3.819 (Å)
$\varepsilon_{\mathrm{Au}-\mathrm{He}}$	0.787 (meV)
$\sigma_{\mathrm{Au}-\mathrm{He}}$	4.342 (Å)

**Table 3:** Variation of energy and momentum accommodation coefficients and MD simulations running time with the pressure in the simulation box for Au–Ar and Au–He pairs.

System	Pressure (MPa)	Number Density (1/nm^3^)	MFP (nm)	EAC	MAC	MD Simulations time (ns) *
Au–Ar	2.75	0.59	2.63	0.874	0.883	20
	1.27	0.27	5.71	0.832	0.846	50
	0.84	0.18	8.57	0.816	0.822	70
	0.42	0.09	17.14	0.783	0.791	100
Au–He	0.21	0.048	58.73	0.048	0.059	60
	0.13	0.029	97.89	0.046	0.057	90
	0.08	0.019	146.84	0.043	0.052	150
	0.04	0.009	293.70	0.042	0.049	250

* The time required to record 100,000 collisions

**Table 4:** Momentum accommodation coefficient in three directions (α_x_, α_y_, α_z_) and energy accommodation coefficients (α_E_) results for Ar and He colliding with Au surface at T = 300 K using gas-wall interactions obtained different mixing rules, as well as existing ab-initio calculations.

System	Pair potential	α_x_	α_y_	α_z_	α_E_
Au–Ar	Ab-initio (Parallel walls)	0.824	0.913	0.832	0.874
	Ab-initio (Molecular beam) [22]	0.40	0.77	0.40	0.56
	Fender Halsey	0.915	0.934	0.913	0.913
	Experimental results: α_E_ = 0.85 [34] ; TMAC(α_x_, α_z_) = 0.893 [35] *				
Au–He	Ab-initio (Parallel walls)	0.036	0.113	0.038	0.048
	Ab-initio (Molecular beam) [22]	0.013	0.046	0.014	0.017
	Fender Halsey	0.245	0.347	0.221	0.069
	Lorentz-Berthelot	0.642	0.748	0.653	0.187
	Experimental result: α_E_ = 0.31 [34]				

* No temperature is reported in reference [35].
